# Integrated Bioinformatics Analysis Reveals Function and Regulatory Network of miR-200b-3p in Endometriosis

**DOI:** 10.1155/2020/3962953

**Published:** 2020-07-29

**Authors:** Wanxue Hu, Qin Xie, Yicong Xu, Xin Tang, Hongbo Zhao

**Affiliations:** ^1^Biomedical Engineering Research Center, Kunming Medical University, Kunming 650500, China; ^2^School of Rehabilitation, Kunming Medical University, Kunming 650500, China; ^3^Yunnan Key Laboratory of Stem Cell and Regenerative Medicine, Kunming 650500, China

## Abstract

**Objective:**

MicroRNAs play vital roles in the development of endometriosis. It is reported that miR-200b-3p is downregulated in endometriosis, although its mechanisms in this disease remain still unclear. Therefore, the purpose of this study was to explore the function and potential regulatory network of miR-200b-3p in endometriosis through database analysis.

**Methods:**

The endometriosis gene expression profiles were downloaded from the GEO database to screen differentially expressed genes (DEGs). The predicted and validated target genes of miR-200b-3p were obtained from miRWalk and miRTarBase database. Then, a comparison was performed between miR-200b-3p target genes and DEGs. GO enrichment and KEGG pathway analysis of the target genes was performed using clusterProfiler package. STRING was used to predict the protein-protein interaction among the proteins encoded by the target genes. Then, TransmiR, LncBase, StarBase, PROMO, and AnimalTFDB were employed to identify interactive transcription factors and lncRNAs of miR-200b-3p.

**Results:**

miR-200b-3p was associated with the transcription factors DNMT1, EZH2, HNF1B, JUN, MYB, ZEB1, and ZEB2 during the pathogenesis of endometriosis. The downstream 110 target genes were involved in the biological processes of positive regulation of MAPK cascade, muscle cell proliferation, organ growth, vasculogenesis, and axon development. KEGG analysis revealed that the main pathways related to miR-200b-3p were microRNAs in cancer, PI3K-Akt signaling pathway, colorectal cancer, and tight junction. In addition, four lncRNAs such as MALAT1, NEAT1, SNHG22, and XIST interacted with miR-200b-3p and were associated with transcription factors FOXP3 and YY1.

**Conclusion:**

The predicted target genes and molecular regulatory network of miR-200b-3p in endometriosis not only revealed its biological function but also provided a valuable guideline for further research.

## 1. Introduction

Endometriosis is a benign gynaecological disease characterized by endometrial glands and stroma located outside the uterine cavity. It affects 10–15% of women in their reproductive age and results in a significantly affected quality of life [1, 2]. Endometriosis symptoms include dyschezia, deep dyspareunia, dysuria, and dysmenorrhea, but the pathogenesis of endometriosis is still largely unknown [3]. Therefore, a better understanding of the molecular mechanism underlying endometriosis progression is of utmost importance to identify novel and more efficient diagnostic predictors for an accurate identification of the disease to use optimal therapeutic strategies.

MicroRNAs (miRNAs) are small, single-strand noncoding RNA molecules containing ~22 nucleotides, which regulate the expression of target genes and control a variety of cellular functions, including apoptosis, proliferation, migration, and invasion [4]. Furthermore, through a combinatorial action of miRNAs, transcription factors (TFs), and long noncoding RNAs (lncRNAs), more complex regulatory networks are often involved in various biological events [5]. Previous microarray studies demonstrated that some miRNAs are aberrantly expressed in endometriosis [6–8]. miR-200b-3p, one of the miRNAs downregulated in endometriosis [9, 10], belongs to the miR-200 family, and it is involved in the regulating of epithelial-to-mesenchymal transition (EMT) in endometriotic cells [11]. However, little is known about the potential function and regulatory network of miR-200b-3p in endometriotic lesions.

Therefore, in this study, an integrated analysis of gene expression profile datasets from GEO database was performed to screen differentially expressed genes (DEGs) in endometriosis. Then, the target genes of miR-200b-3p and the network it regulates were investigated using bioinformatics tools. Altogether, our results showed that miR-200b-3p might play critical roles in the pathology of endometriosis.

## 2. Materials and Methods

### 2.1. Expression Profile in Endometriosis

Two endometriosis microarray data sets were downloaded from the GEO website (http://www.ncbi.nlm.nih.gov/geo/). GSE7305 microarray data contained 10 endometriosis tissues and 10 normal endometrial tissues [12]. GSE7307 included 18 endometriosis samples and 23 healthy endometrial samples. In both datasets, the disease samples are ovarian endometriosis, which is the most frequently occurring type. The raw data were first corrected by RMA (Robust Multichip Averaging) algorithm and standardized together with log2 [13]. The DEGs were filtrated using the limma package [14]. The screening criteria were set at a false discovery rate-adjusted *p* value (FDR) <0.05 and fold change >1.5. DEGs that appeared in both sets of data were selected for subsequent analysis.

### 2.2. Prediction of Target Genes

The miRWalk 2.0 tool (http://zmf.umm.uni-heidelberg.de/mirwalk2/) [15], which is linked to other 11 online prediction databases, was used to predict the putative target genes of miR-200b-3p. Only mRNA targets predicted by four miRNA prediction databases (miRWalk, miRanda, RNA22, and TargetScan), and in common, among them were considered for further analysis. Then, the validated mRNA targets of miR-200b-3p were downloaded from the miRTarBase database [16] and combined with predicted targets. Finally, the target genes of miR-200b-3p in endometriosis were obtained by comparing miR-200b-3p candidate target genes and DEGs from GEO.

### 2.3. Functional Enrichment Analysis of Target Genes

The clusterProfiler package [17] was used to evaluate GO functional (biological processes) and KEGG pathway analysis on the target genes. FDR <0.05 was used as the cutoff criterion.

### 2.4. Protein-Protein Interaction (PPI) Analysis of Target Genes

Target genes were submitted into STRING (https://string-db.org/) [18] to analyse the interaction between the encoded proteins. The pairs with combined scores >0.4 were used to construct the PPI network; then, the Cytoscape software was used for visualizing the network [19].

### 2.5. Prediction of miR-200b-3p TFs

TransmiR v2.0 is a database for TF-miRNA regulations, which covers 623 TFs and 785 miRNAs and contains 3730 literature-curated TF-miRNA regulations [20]. Therefore, the TFs linked with miR-200b-3p were predicted using TransmiR v2.0, and then, the ones included into the DEGs of endometriosis were screened.

### 2.6. Prediction of the Candidate lncRNAs for miR-200b-3p

LncBase Experimental v2 is an online software for experimental validation and computational derivation of the relationship between miRNA and lncRNA, which was used to predict lncRNAs associated with miR-200b-3p [21]. The StarBase database is based on CLIP-seq data and tumour samples (14 cancer types, >6000 samples), and analyses the interaction between lncRNA, miRNA, ceRNA, RBPs, and mRNA [22]. miRNA-lncRNA module in StarBase was used to screen lncRNAs binding to miR-200b-3p, and the results were compared to the ones obtained with LncBase Experimental v2.

### 2.7. Prediction of lncRNA Associated TFs

PROMO is a program that predicts TF binding sites in related sequences from a species through the TRANSFAC database [23]. AnimalTFDB is a source providing annotation and prediction of animal TFs from 97 animal genomes and contains 125,135 TF genes and 80,060 transcription cofactor genes [24]. The two databases were used to predict the TFs of lncRNAs and then to take the intersection of the results.

### 2.8. Construction of the miR-200b-3p Network Regulating Endometriosis

On the basis of the genomic information that was collected, the potential regulatory network of miR-200b-3p in endometriosis was constructed.

## 3. Results

### 3.1. Identification of DEGs

As regards DEG analysis, GSE7305 contained 4223 upregulated probes and 3830 downregulated probes in endometriosis, while GSE7307 contained 2360 upregulated probes and 2200 downregulated probes. After comparison, 2105 common upregulated probes corresponding to 1264 genes and 1867 common downregulated probes corresponding to 1269 genes were obtained.

### 3.2. Identification of miR-200b-3p Target Genes in Endometriosis

**Figure 1:**
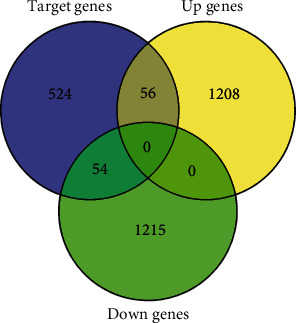
Venn diagram of the intersection analysis between miR-200b-3p target genes and DEGs in endometriosis.

A total number of 482 predicted miR-200b-3p target genes that appeared in the four prediction databases were obtained. A total of 186 validated target genes associated with miR-200b-3p were obtained from the mirTarBase. The two sets were taken together, and 634 candidate target genes of miR-200b-3p were obtained and compared with the DEGs obtained from the microarray data analysis. Finally, 110 target genes of miR-200b-3p in endometriosis, including 56 upregulated genes and 54 downregulated genes, were obtained (Figure 1).

### 3.3. GO and KEGG Pathway Enrichment Analysis of the Target Genes

**Figure 2:**
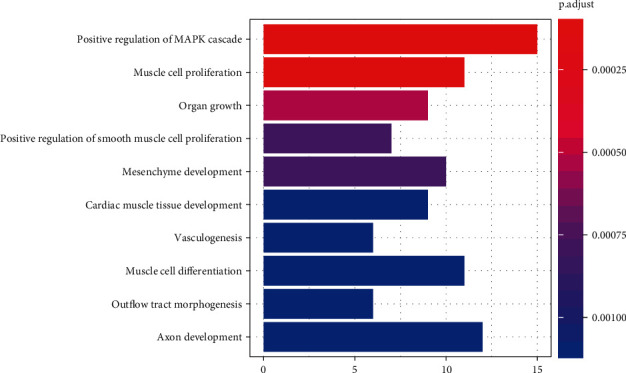
Top 10 significantly enriched biological processes identified by GO analysis of miR-200b-3p target genes.

GO analysis showed that the target genes were mainly enriched in biological processes such as positive regulation of MAPK cascade, muscle cell proliferation, organ growth, vasculogenesis, and axon development (Figure 2). In addition, KEGG pathway analysis indicated that these target genes were mainly involved in pathways of microRNAs in cancer, PI3K-Akt signaling pathway, colorectal cancer, and tight junction (Table 1).

### 3.4. PPI Network Construction

**Figure 3:**
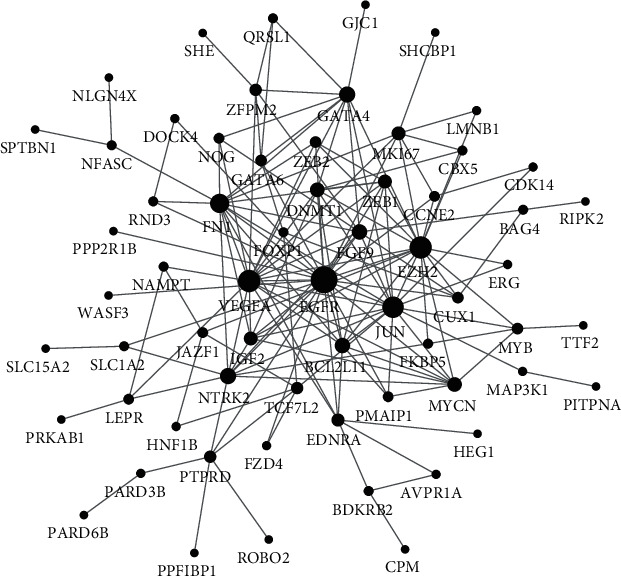
Protein-protein interaction network analysis of the target genes. The PPI network consisting of 63 nodes and 155 edges was constructed using the target genes. The size of the circle represents the degree of a node.

STRING was used to extract the proteins expressed by the target genes that can interact with others. A total of 63 target genes were filtered into the PPI network, containing 63 nodes and 155 edges (Figure 3). The NetworkAnalyzer app in Cytoscape was used to calculate the node degree. The top 5 genes were the following: EGFR, EZH2, VEGFA, JUN, and FN1 with a degree >15; thus, they might be regulated by miR-200b-3p and play important roles in the progression of endometriosis.

### 3.5. TFs Associated to miR-200b-3p

A total of 75 TFs associated with miR-200b-3p were obtained from the TransmiR v2.0 database. Then, DNMT1, EZH2, HNF1B, JUN, MYB, ZEB1, and ZEB2 were found as TFs related to endometriosis by interacting with target genes of miR-200b-3p.

### 3.6. miR-200b-3p-lncRNAs Association

The lncRNAs associated with miR-200b-3p were predicted by LncBase Experimental v2 and StarBase databases, and the lncRNAs overlapping in both the databases were selected, such as MALAT1, NEAT1, SNHG22, and XIST. Thus, the mutual regulation of these four lncRNAs and miR-200b-3p might play an important role in the development of endometriosis.

### 3.7. Identification of lncRNA Associated TFs

PROMO and AnimalTFDB were used to predict the TFs that could be associated with four lncRNAs (MALAT1, NEAT1, SNHG22, and XIST) in the upstream 2 kb promoter sequence. The predicted intersections resulted in two TFs, such as FOXP3 and YY1.

### 3.8. Functional Regulatory Network of miR-200b-3p in Endometriosis

**Figure 4:**
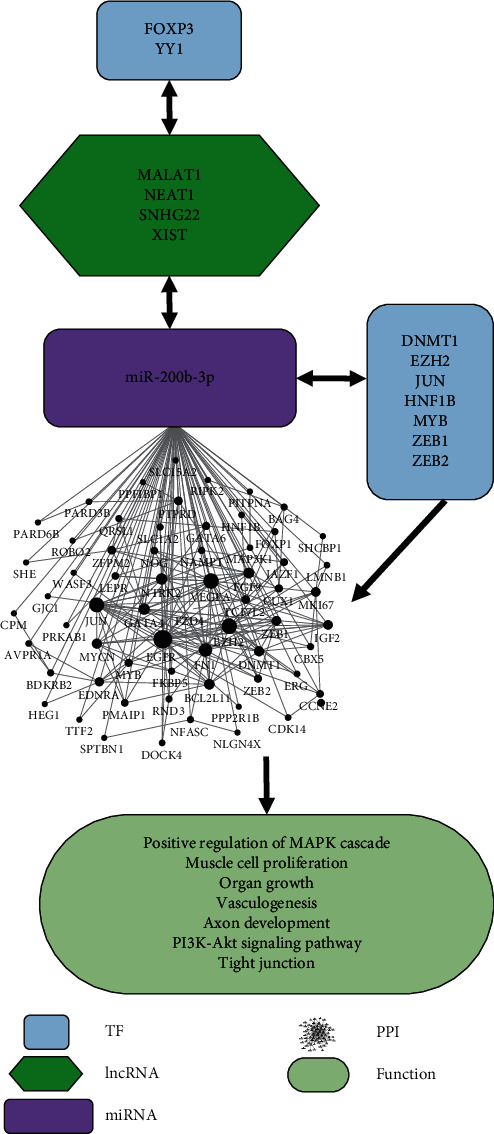
Predicted functional regulatory network of miR-200b-3p in endometriosis.

From the above prediction results, a miR-200b-3p network regulating endometriosis was constructed (Figure 4).

## 4. Discussion

Although endometriosis is a benign inflammatory disease, some of its characteristics are comparable to the ones of malignant tumours, such as abnormal cell migration, implantation, invasion, and angiogenesis. Several studies indicated that miR-200b-3p is decreased in endometriosis, but its specific pathogenesis in this disease is not yet clear. In this study, the miR-200b-3p network that regulates endometriosis was constructed by integrating microarray data sets and the target pair information from several interactome databases. The four relationships considered in this network were the following: (1) the regulatory pair of miR-200b-3p and target genes, (2) the regulatory pair of miR-200b-3p and TF genes, (3) the regulatory pair of miR-200b-3p and lncRNA genes, and (4) the regulatory pair of lncRNA genes and TF genes. A total of 110 target genes, 7 TF genes, and 4 lncRNAs associated with miR-200b-3p were identified in endometriosis. The functions of the target genes were analysed, and the enriched GO terms were positive regulation of MAPK cascade, muscle cell proliferation, organ growth, vasculogenesis, and axon development. These results suggested that the identified miR-200b-3p target genes in endometriosis and the associated regulators (TFs and lncRNAs) might play an important role in the induction of endometriosis.

By integrating the information from gene expression profiles, the target genes of miRNA can be found more accurately; thus, the genes regulated by miR-200b-3p in endometriosis can be also discovered. A total of 110 target genes of miR-200b-3p were screened, and many of them play important roles in the development of endometriosis. They included FOXP1, GATA6, JUN, and EGFR involved in muscle cell proliferation [25–28]; NTRK2, ZFPM, and VEGFA involved in angiogenesis [29, 30]; and DNMT1, EZH2, FOXP1, VEGFA, ZEB1, and ZEB2 involved in EMT [31, 32]. Recent studies found that EMT plays an important role in the occurrence and development of endometriosis [33]. The miRNA-200 family is a recognized epithelial cell marker and is a major regulator of EMT [34]. Other studies showed that miR-200b-3p downregulates ZEB1 and ZEB2 in endometriosis patients and participates in the feedback regulation of EMT [35], which more easily promotes cell invasion and metastasis. Our results showed that EGFR, EZH2, VEGFA, JUN, and FN1 are important nodes in the target genes PPI network. These results indicated the high accuracy in predicting target genes, and they also proved that miR-200b-3p had important regulatory effects on endometriosis. In addition, some predicted genes related to endometriosis were not discovered during the research process and reported. Although these genes have not been fully studied and verified, they might become important targets in the treatment of endometriosis.

miRNAs not only regulate the development of several diseases by interacting with protein-coding genes but also promote or inhibit cell proliferation, apoptosis, and metastasis by interacting with lncRNAs. The lncRNAs associated with miR-200b-3p were predicted by LncBase and StarBase databases and identified 4 overlapping lncRNAs (MALAT1, NEAT1, SNHG22, and XIST). Therefore, these four lncRNAs might have a mutual regulation with miR-200b-3p. Liang et al. found that the expression of MALAT1 is significantly upregulated in ectopic endometrial tissue and is negatively correlated with the expression of miR-200c [36], suggesting that MALAT1 is a target of miR-200c in primary endometrial stromal cells. Since miR-200c and miR-200b are both belonging to the miRNA-200 family, this could mean that MALATI is a potential target of miR-200b-3p regulating endometrial stromal cells. However, the relationship between these four lncRNAs and miR-200b needs further experimental verification.

The common predicted TFs of the four lncRNAs were FOXP3 and YY1. FOXP3 is essential in the development and function of regulatory T cells. Ectopic Foxp3 expression can phenotypically convert effector T cells to regulatory T cells [37]. A recent research found that endometriosis is related to changes in Treg and Th17 cells in the pelvis, leading to the survival and implantation of ectopic endometrial lesions [38]. The percentage of CD25^+^FOXP3^+^Treg cells is significantly increased in the peritoneal fluid of women with endometriosis [39]. Furthermore, the combination of FCRL3 and FOXP3 genotypes increases the risk of endometriosis, as observed in the Brazilian population samples [40]. YY1 is a TF with diverse and complex biological functions and has a role in controlling X chromosome inactivation (XCI) [41]. YY1 binds directly the Xist 5′ region to trigger the activity of the Xist promoter [42], and loss of YY1 prevents Xist upregulation during the initiation and maintenance of X-inactivation [43]. Furthermore, YY1 deletion or disruption in activated female T and B cells disrupts Xist RNA localization to the inactive X with minimal impact on Xist expression, suggesting that YY1 may regulate XCI maintenance through the mediation of Xist RNA localization in lymphocytes [44]. In addition, endometriosis is an estrogen-dependent disease characterized by chronic inflammation and clinically by chronic pelvic pain. Current evidence suggests that mast cells and microglia are considered coordinators of peripheral and central inflammatory processes, and may trigger and sustain the persistent inflammatory process present in the pathogenesis of endometriosis [45]. Han et al. confirmed that YY1 upregulates NEAT1 in the oxygen-glucose deprivation/reoxygenation injury of microglial cells [46].

In conclusion, based on the results of various bioinformatics methods, our hypothesis is that miR-200b-3p is associated with TFs such as DNMT1, EZH2, HNF1B, JUN, MYB, ZEB1, and ZEB2 in the occurrence and development of endometriosis, regulating downstream target genes, and it is also regulated by four lncRNAs. Furthermore, these four lncRNAs interact with the TFs FOXP3 and YY1. Although the prediction of the molecular regulatory network of miR-200b-3p by bioinformatics needs further experiments to be confirmed, these predictions may represent a valuable guideline in the mechanism triggering endometriosis.

## Figures and Tables

**Table 1:** KEGG pathway enrichment analysis on 110 target genes of miR-200b-3p.

Pathway ID	Pathway name	FDR	Included genes
hsa05206	MicroRNAs in cancer	0.0073	BCL2L11, FOXP1, ZEB2, ZFPM2, CCNE2, DNMT1, EGFR, EZH2, VEGFA, ZEB1
hsa04151	PI3K-Akt signaling pathway	0.0111	BCL2L11, FN1, NTRK2, PPP2R1B, CCNE2, EGFR, FGF9, IGF2, MYB, VEGFA
hsa05210	Colorectal cancer	0.0162	BCL2L11, JUN, TCF7L2, EGFR, PMAIP1
hsa05215	Prostate cancer	0.0212	ERG, TCF7L2, CCNE2, EGFR, ZEB1
hsa04530	Tight junction	0.0337	GATA4, JUN, PPP2R1B, MAP3K1, PARD6B, PRKAB1
hsa05165	Human papillomavirus infection	0.0493	FN1, FZD4, PPP2R1B, TCF7L2, CCNE2, EGFR, PARD6B, VEGFA

## Data Availability

The microarray raw data used in this study are freely available from GEO datasets GSE7305 and GSE7307. In addition, the datasets used for network construction were obtained from the tool websites.
